# A Novel Flexible Liquid Metal Microheater with a Textured Structure

**DOI:** 10.3390/mi15010075

**Published:** 2023-12-29

**Authors:** Yuqing Li, Huimin Zhang, Zi Ye, Mingyang Liu, Wei Liu, Zhenming Li, Lin Gui

**Affiliations:** 1 Key Laboratory of Cryogenic Science and Technology, Technical Institute of Physics and Chemistry, Chinese Academy of Sciences, 29 Zhongguancun East Road, Haidian District, Beijing 100190, China; liyuqing201@mails.ucas.ac.cn (Y.L.); zhanghuimin19@mails.ucas.ac.cn (H.Z.); yezi_1992@mail.ipc.ac.cn (Z.Y.); 2 School of Future Technology, University of Chinese Academy of Sciences, Beijing 100039, China; 3 School of Engineering Science, University of Chinese Academy of Sciences, Beijing 100039, China; 4 Energy Storage and Novel Technology of Electrical Engineering Department, China Electric Power Research Institute, Beijing 100192, China; liumingyang@epri.sgcc.com.cn (M.L.); liuwei3@epri.sgcc.com.cn (W.L.); lizhenming@epri.sgcc.com.cn (Z.L.)

**Keywords:** liquid metal microheater, textured structure, ventilating channels, flexibility

## Abstract

In this paper, we propose a novel liquid metal microheater utilizing a textured structure. This microheater effectively solves the problem of the liquid metal in the PDMS flow channel fracturing at a certain temperature and significantly increases the maximum operating temperature that can be achieved by the current liquid metal microheater. Experimental results demonstrate that this new structured microheater can achieve a maximum operating temperature exceeding 300 °C. To explain the performance improvement and the reasons behind liquid metal fracture, corresponding experiments were conducted, and explanations were provided based on the experimental results. Subsequently, we verified the mechanical flexibility of the microheater and found that it exhibits excellent tensile and bending resistance. Finally, utilizing its good mechanical flexibility, the microheater was successfully attached to the side wall of a cup, resulting in the boiling of water.

## 1. Introduction

With the rapid development of microelectromechanical systems (MEMS) technology, microelectromechanical sensors are being gradually developed in the direction of miniaturization, integration, and intelligence. In microsystems, microheaters have the role of providing a temperature field and precise temperature control for the system. At present, the applications of microheaters on MEMS include flow meters [1], gas sensors [2,3,4], temperature sensors [5], biology-related applications [6,7,8], etc. Among them, flexible microheaters that can fit on curved surfaces and apply thermal stimulation in a small area have shown unique applications in many emerging fields such as wearable electronics and portable medical devices [9,10].

Solid metal thin film resistance microheaters are the most common choice for flexible microheaters today. Solid metal thin films are made by physical vapor deposition (PVD) [11], chemical vapor deposition (CVD) [12], electrochemical deposition [13], ultrasonic spray pyrolysis system (USPS) [14], etching [15], and other means to make solid metals (Pt [16,17], Au [18], Ti [19], etc.) on the surface of flexible substrates, such as polyimide (PI) films [20] and polyethylene terephthalate (PET) sheets [21]. Such microheaters with solid metal thin-film resistors are not only very conducive to system integration and miniaturization but also have very stable electrical, thermal, and mechanical properties, and the fabrication processes are complex and costly. Therefore, non-metallic materials with excellent thermal and electrical properties, such as carbon nanotubes (CNT) [22] and graphene [23], have become popular materials for making flexible microheaters in recent years. Jia et al. [22] used a simple solution process with a spray-coating method to fabricate the electrothermal heating film-based CNTs on flexible PET substrates. They demonstrated that these heating films have high performance with over 80% transmittance at 550 nm and reach a steady-state temperature of up to 75.5 °C at an applied voltage of 30 V. Wang et al. [23] developed a flexible graphene microheater that can be used for high efficient temperature control and has outstanding long-term performance using ultrafast laser ablation. Since these non-metallic materials are usually formed directly on the surface of flexible substrates to form the heating element, this type of microheater has good mechanical flexibility. However, the biggest drawback of these microheaters is the maximum temperature that can be reached is much lower than that of the metal film microheaters.

To simplify the fabrication method of microheaters, reduce the cost, and ensure a certain heating temperature, Wu et al. [24] proposed a new method to fabricate microheaters using a microfluidic chip. They injected silver paint into the polydimethylsiloxane (PDMS) microchannels and then evaporated the organic solvent to form a conductive microheater wire composed of silver particles in the PDMS microchannels. Although the PDMS-based microfluidic chip is flexible, silver is a solid metal, and the overall mechanical flexibility of the microheater is not good. However, the widespread use of room-temperature liquid metals (RTLMs), especially gallium-based alloys, in recent years has effectively solved this problem. Liquid metals have the fluidity of fluids and excellent electrical and thermal conductivity [25,26]. The fabrication of flexible microheaters by injecting liquid metals into PDMS microchannels is not only simple and fast but also allows the fabrication of “thick” electrodes that are not easily fabricated by other processes. However, the fracture of liquid metals at low temperatures (about 75 °C) has severely limited the application of these microheaters [27]. Initially, two hypotheses were proposed for this fracture phenomenon in liquid metals, one is the PDMS thermal expansion hypothesis [27] and the other is the electromigration hypothesis [28,29]. But neither of these two hypotheses gave very strong experimental evidence to prove their reliability. Later, Zhang et al. [30] proposed a new conjecture. They suggested that the fracture of liquid metals inside the PDMS microchannels is due to the thermal expansion of the tiny gas cavities inside microchannels that were originally present and eventually caused the fracture of liquid metals. To test the conjecture, they designed a new microheater structure, in which ventilation air channels were added on both sides of the liquid metal heating channel to release the expanding gas. Experimental results show that this new structure does substantially increase the upper-temperature limit (about 220 °C) that the liquid metal microheater can reach. However, the microheater still has some drawbacks, and the most important drawback is that the presence of PDMS micro-posters greatly limits the length of the microheater (the maximum length of the liquid metal heating wire is only 6 cm), because the longer the microheater, the more difficult it is to ensure that the liquid metal does not leak from the micro-posters during the injection.

In this study, we designed a new structure of a liquid metal microheater to solve the above problem based on the conjecture of Zhang et al. [30]. We used a PDMS film with a textured structure as the bottom membrane of the microheater for venting and fabricating a liquid metal microheater with a length of about five times that of Zhang et al.’s [30] microheater. The experimental results showed that the microheater can reach a higher temperature limit (over 300 °C). We also compared the experimental results with Zhang et al. [30] and found another reason for the fracture of the liquid metal microheater at this upper-temperature limit and explored the reason through further experiments. In addition, to test the mechanical flexibility of the microheater, tensile and bending experiments were performed on the microheater. Finally, the liquid metal microheater with the textured structure was attached around the side of the cup wall to heat the water. It heated water from room temperature to boiling within 30 min. This demonstrated not only the good heating performance of the microheater but also its good mechanical flexibility to be attached to any curved surfaces. Therefore, in the future, it can be widely used in various flexible heating applications.

## 2. Materials and Methods

**Figure 1:**
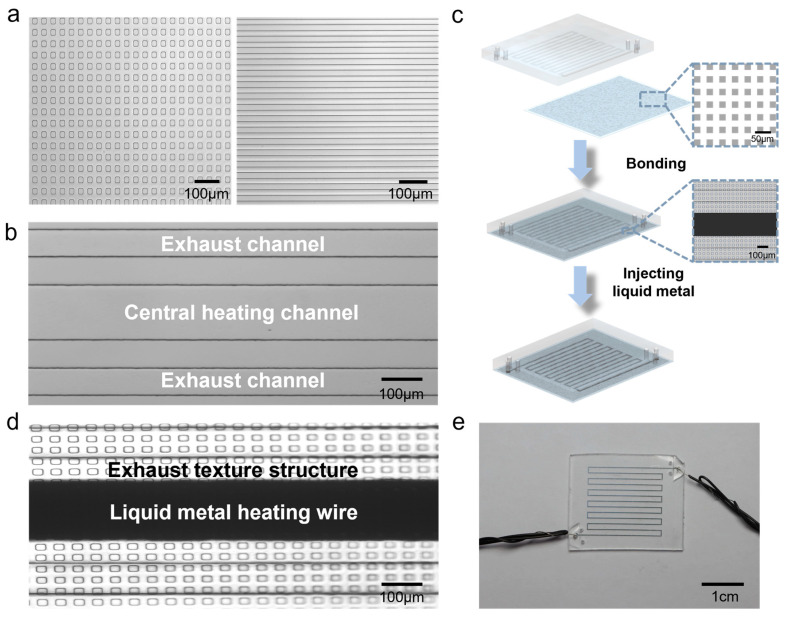
Structure of the liquid metal microheater: (**a**) textured structure of the bottom layer, (**b**) microchannel structure of the top layer, (**c**) chip-making process, (**d**) microstructure after injection of liquid metal, and (**e**) physical image of a microheater.

Firstly, we fabricated a silicon wafer with textured structures, referred to as texture wafer, using the method proposed in our previous study [31]. This texture-like structure consists of many recessed exhaust channels only a few micrometers high and wide. Due to the high surface tension of liquid metal, the structure can prevent liquid metal from seeping into the texture-like structure during the injection while at the same time letting the air to release from it. Then, we utilized a standard soft lithography process to fabricate heater structures with another silicon wafer, referred to as microheater wafer. A layer of liquid PDMS (Sylgard 184 silicone elastomer kit, Dow Corning, Midland, MI, USA) film about 200 μm thick was spin-coated on texture wafer using a spin coater at a speed of 200 rpm. On the microheater wafer, we poured 10 g of liquid PDMS, then waited for its natural leveling on the wafer. Subsequently, two silicon wafers were placed on the hot plate at 75 °C and baked for 2 h to cure the PDMS. Then, the PDMS film with textured structure was peeled off from texture wafer as the exhaust substrate film for the microheater, as shown in Figure 1a. The PDMS block with microheater structure was peeled off from microheater wafer as the main structure layer of the microheater, as shown in Figure 1b. It consisted of a central heating channel and two parallel exhaust channels, with a distance of 100 μm between the heating channel and the exhaust channels. The exhaust channels are used to collect air escaping through the narrow textured structure, enabling its fast release to the external area of the chip. Afterward, we punched holes at each end of the three channels on the PDMS block and bonded it to the substrate after standard air plasma treatment. Finally, we used a syringe to inject EGaIn into the heating channel and inserted copper wires into the holes at the ends of the heating channels to establish contact with EGaIn, thus completing the fabrication process (Figure 1c). The local structure of the microheater after the injection of liquid metal is shown in Figure 1d, and the physical appearance of the microheater is displayed in Figure 1e. The x-section of the microheater under the SEM is shown in Figure S1. In addition, we fabricated traditional liquid metal microheaters without air release effect using blank PDMS film instead of textured PDMS film following the same process for later comparison.

## 3. Experimental Principle and Procedure

The liquid metal microheater works using Joule’s law of heating. A derivative expression of Joule’s law can be expressed as Equation (1), where P is the thermal power of the microheater, U is the voltage applied across the liquid metal heating wire, and R is the resistance of the heating wire.

(1) $$P={\frac{U}{R}}^{2}$$

**Figure 2:**
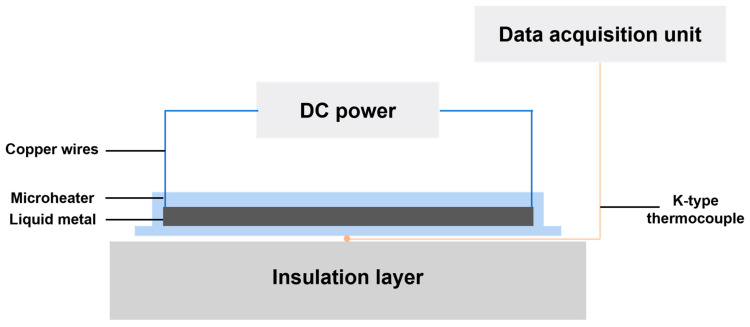
Schematic experimental setup.

Therefore, to verify the improvement of microheater performance by textured structure, we conducted experiments to compare it with the microheater without textured structure. Based on the derived equation of Joule’s law, we built the experimental platform as shown in Figure 2. A DC power (DH1720A, DaHua Power, Beijing, China) was connected to copper wires at both ends of the microheater to apply voltage. A standard K-type SMD thermocouple was placed beneath the microheater substrate to measure the temperature. The thermocouple-equipped microheater was placed on an insulation layer to reduce temperature interference below the thermocouple. Data from the measurement process were collected in real time with Agilent 34972A (Agilent, Santa Clara, CA, USA) and saved via a laptop.

## 4. Results and Discussion

### 4.1. Experimental Results

We compared the performance of the microheater fabricated using two different textured structures, as illustrated in Figure 1a, and microheaters fabricated using blank PDMS (no texture). For the comparative experiments, six kinds of microheaters with different parameters were selected. These six microheaters had a total of three bottom film types (parallel straight texture, a square texture, and a blank PDMS). Each bottom film had two liquid metal channel widths of 200 μm and 400 μm, respectively. In addition, the length and height of the liquid metal channel were the same, 30 cm and 30 μm, respectively. The initial temperatures of the tests were all 27.5 °C. The maximum achievable temperature with and without the exhaust textured structure was tested for liquid metal channels with widths of 200 μm and 400 μm. During the experiment, the voltage applied to the ends of the liquid metal heating wire increased in steps of 0.4 V. The microheater was stabilized at each voltage value for 5 min to ensure thermal equilibrium between the microheater and the environment, allowing the temperature of the microheater to reach a steady state.

**Figure 3:**
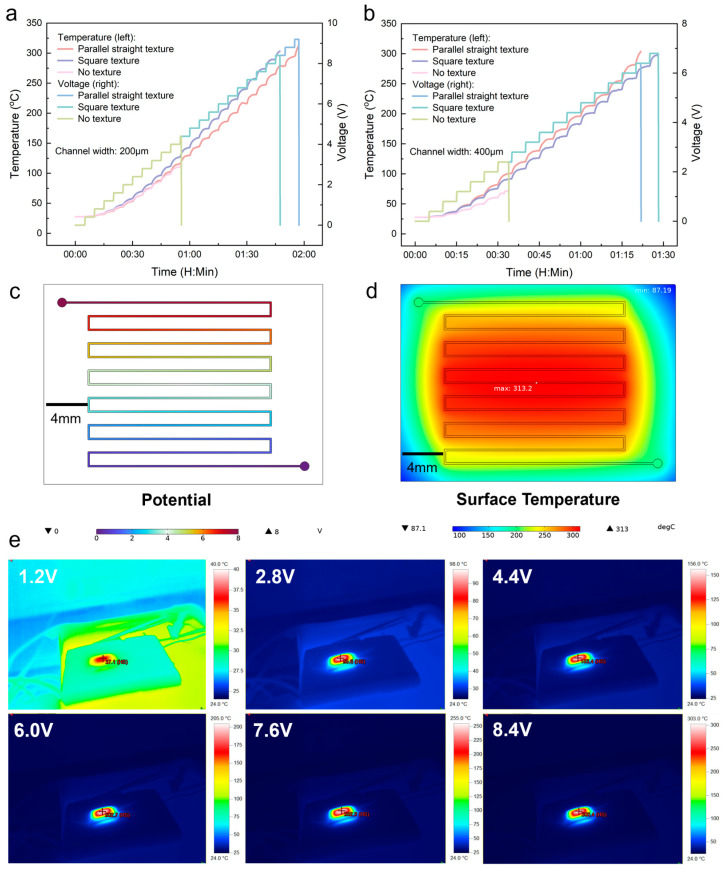
Microheater performance testing and verification. Real-time temperature and voltage profile of microheater: (**a**) channel width of 200 μm, (**b**) channel width of 400 μm, (**c**) potential distribution on the liquid metal heating wire, (**d**) temperature field distribution on the plane of the microheater, and (**e**) infrared imaging of microheaters at different voltages.

The experimental results are shown in Figure 3a,b. Figure 3a shows the real-time temperature–voltage curves of microheaters with different substrate structures for a liquid metal channel width of 200 μm. For a more intuitive comparison, we summarize the results in Table 1. From the table, we can observe that the microheater fabricated using parallel straight texture exhibits the best performance. It reaches a high temperature of 314.2 °C before experiencing rupture at 9.2, and its maximum heating rate can reach 0.1 °C/s. The next best performer is the square textured structure, which ruptures at 8.4 V with a temperature of 303.1 °C. The microheater fabricated without any structured substrate performs the worst, rupturing at only 4.4 V (temperature of 115.5 °C). The results of the temperature–voltage real-time curves for the channel width of 400 µm shown in Figure 3b are similar to those for 200 µm, with both showing the best performance for the parallel textured structure and the worst performance for the unstructured one. All three microheaters (1. parallel straight texture, 2. square texture, and 3. no texture) rupture at different voltage levels, i.e., 6.4 V, 6.8 V, and 2.4 V, respectively, and the corresponding temperatures are 303.8 °C, 298.7 °C, and 72.1 °C. In addition, we conducted simulations and infrared imaging tests on the heating process of the microheater with a channel width of 200 μm. The results are shown in Figure 3c–e, respectively. From the figures, we can see that both the experimental results of simulation and infrared imaging are basically consistent with those measured using thermocouples, further confirming the accuracy of our measurements. The variation of temperature distribution throughout the heating process obtained by simulation is shown in Supplementary Material Video S1, and the infrared imaging of the microheater throughout the heating process is shown in Supplementary Material Video S2. After confirming that the textured structure enhances the performance of the liquid metal microheater, we also experimentally verified that the exhaust channels on both sides of the center heating channel, for collecting the escaping air, also contribute to this performance enhancement (the test results are shown in Figure S2).

**Figure 4:**
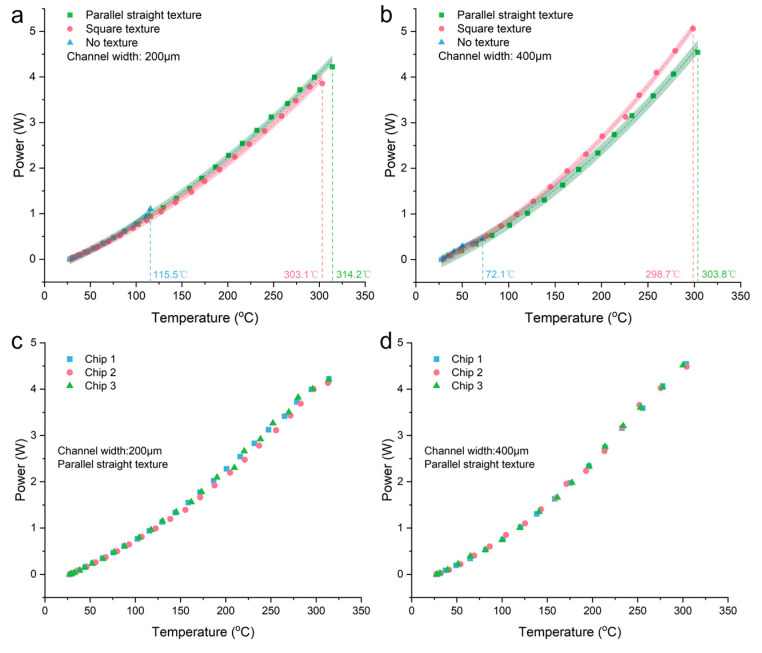
The power–temperature curve of the microheater: (**a**) channel width of 200 μm and (**b**) channel width of 400 μm. Replicate experiments for microheaters with parallel straight textures: (**c**) channel width of 200 μm and (**d**) channel width of 400 μm.

To visually observe the performance advantages of heaters made with textured structures compared to those made with blank PDMS film, we plotted the power–temperature relationship when applying voltage to three types of microheaters. From Figure 4a,b, it can be seen that the heaters with textured structures can achieve much higher power and temperature compared to the heaters made with blank PDMS films, and different textured heaters can reach similar temperatures at the same power level. However, comparing Figure 4a,b, it can be observed that for different widths of liquid metal channels, the heater with a width of 200 µm achieves a slightly higher temperature than the one with a width of 400 µm at the same power level. We believe this is because when the width of the liquid metal heating element increases, the liquid metal can contact a larger area of the textured structure, which means a larger contact area with ambient air. When voltage is applied to the ends of the liquid metal wire and Joule heat is generated, the larger heat exchange area with the air results in a slightly lower temperature than can be achieved. In addition, to prove the accuracy and reproducibility of our measured data, we chose the microheater with parallel linear textured structure that has the best performance under two channel widths to conduct three repetitive experiments, and the results obtained are shown in Figure 4c,d, respectively. Through the overlap of the scatter distributions, we can find that for multiple chips with the same kind of structure, the performance they exhibit is roughly the same, indirectly proving the reproducibility and accuracy of our test results.

### 4.2. Microheaters with Textured Structure vs. Microheaters with Parallel Ventilating Side Channels

**Figure 5:**
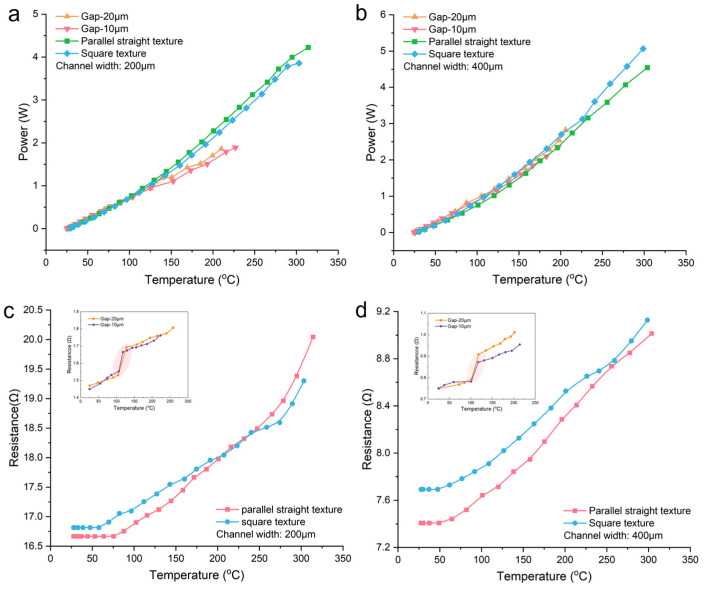
Microheater comparison and power comparison: (**a**) 200 µm width and (**b**) 400 µm width. Resistance comparison (the data in the upper left vignette are from ref. [30]): (**c**) 200 µm width and (**d**) 400 µm width.

After establishing the significant performance improvement of microheaters using textured structures compared to traditional microheaters, we conducted a comparison between these microheaters and microheaters with parallel ventilating side channels, as shown in Figure 5. It illustrates the variation of power and resistance of liquid metal microheaters with temperature as the applied voltage increases. As shown in Figure 5a,b, the power and temperature that can be achieved with the texture microheater are much higher than those of the microheater with parallel ventilating side channels, regardless of whether the liquid metal channel width is 200 µm or 400 µm. The microheaters with parallel ventilating side channels reach a maximum temperature just above 200 °C, while microheaters with textured structures can achieve temperatures around 300 °C, an increase of approximately 100 °C. Figure 5c,d depict the variation of the resistance of liquid metal microheaters with temperature. We can see that the liquid metal resistance of both microheaters increases gradually as the temperature increases. However, there is a significant difference between the two. In the insets of Figure 5c,d, it can be observed that microheaters with parallel ventilating side channels experience a sharp increase in resistance at a certain temperature (highlighted by shaded circles), and the slope of the resistance curve after the abrupt change is greater than the one before. Zhang et al. [30] suggested that this phenomenon is caused by the appearance of voids in the liquid metal microchannel, leading to a decrease in cross-sectional area and a significant change in resistance, followed by the growth of subsequent voids, resulting in an increased slope of the resistance curve. On the other hand, the resistance of microheaters utilizing textured structures changes continuously with temperature, suggesting that there may be no occurrence or growth of voids within the microheater during the heating process until final rupture.

### 4.3. Analysis and Discussion

From the analysis of the results at the end of the previous section for the microheater utilizing a textured structure compared to the microheater with parallel ventilating side channels, we propose a hypothesis that our designed microheater may not have the process of void generation and growth internally during the heating process. In addition, another reason supporting this hypothesis is that during the testing of microheaters with widths of 200 µm and 400 µm, we found that the highest temperatures they can reach are very close. This is quite different from the conclusion of Zhang et al. [30], who found that the microheater with a width of 400 µm could reach a significantly lower maximum temperature compared to the one with a width of 200 µm. According to their analysis of the fracture reason for microheaters, this phenomenon is highly reasonable. This is because the microheater with a width of 400 µm requires nearly twice the amount of liquid metal injection compared to the one with a width of 200 µm. Therefore, the amount of air trapped inside the liquid metal is larger in a wider channel, resulting in a higher probability of cavity expansion and liquid metal fracture caused by air expansion due to thermal expansion. Hence, we believe that the fracture of our designed microheater using textured structure is not caused by the thermal expansion of internal air that leads to the fracture of liquid metal.

**Figure 6:**
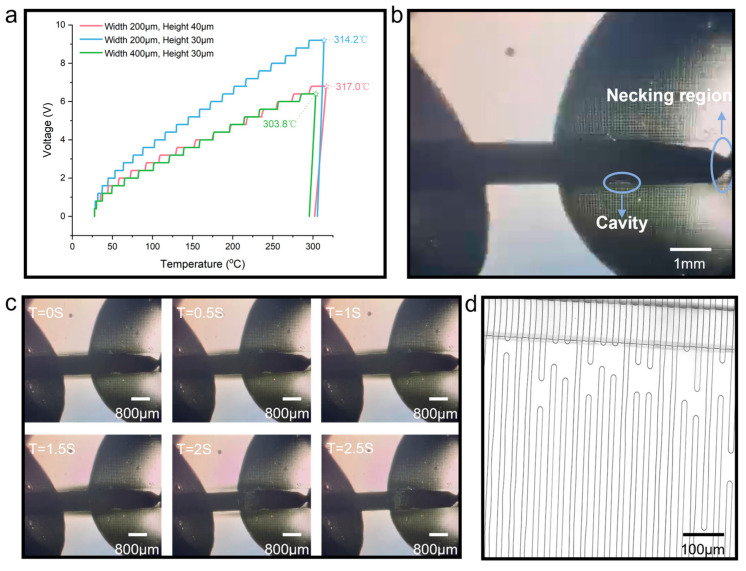
(**a**) Performance comparison of microheaters with different widths and heights. (**b**) Full view of the microheater under the microscope. (**c**) Time series diagram of liquid metal fracture process. (**d**) Textured structure after liquid metal fracture under the microscope.

To test this hypothesis, we conducted a series of comparative experiments. Three sets of microheaters with the same channel height, different widths, and the same width and different heights were selected for testing (all microheaters have identical parallel straight textured structures on the bottom film). The voltage applied to the ends of the liquid metal heating wire increased in steps of 0.4 V, and each voltage value was stabilized for 5 min. The experimental results are shown in Figure 6a. We can observe that for microheaters with a channel width of 200 µm, the height of channels has little effect on the maximum temperature they can reach (the microheater with a channel height of 30 µm can reach a maximum temperature of 314.2 °C, while the one with a height of 40 µm can reach a maximum temperature of 316.9 °C.). However, for microheaters with the same channel height but different widths, the microheater with a wider channel achieves a slightly lower maximum temperature (the microheaters with two different channel widths have a temperature difference of approximately 10 °C in their maximum temperatures). Through this set of comparative experiments, we are more confident that the fracture mechanism of the microheater made using textured structures is no longer due to the thermal expansion of air. Therefore, it requires further exploration and discussion.

We designed a microheater with a length of only 6 mm using textured structures so that its entire appearance could be observed under a microscope. We placed the microheater under the microscope and applied voltage to its two ends. The initial state of the microheater under the microscope is shown in Figure 6b. It is visible that there is a cavity and a necking region in the initial state of the microheater. When the applied voltage reached 5.2 V, the liquid metal microheater fractured. The Supplementary Material Video S3 demonstrates the process of liquid metal microheater fracture. The time sequence of the fracture of the liquid metal microheater is shown in Figure 5c. From this time sequence graph, we can observe a significant thermal expansion of the PDMS at the moment of fracture. For Sylgard 184, we found that the thermal expansion coefficient of PDMS is 340 ppm °C^−1^ [32] in the official website’s datasheet. This means that when PDMS is heated from room temperature to around 300 °C, its volume will expand by about 9.18%. At T = 2 s, the thermal expansion of PDMS reaches its maximum and a large gap appears between the liquid metal inside the channel and the channel boundary; therefore, the liquid metal cannot fill the entire channel and leads to fracture. However, at the moment of fracture, as the temperature of the microheater rapidly decreases, the thermal expansion phenomenon of PDMS also disappears, as shown in Figure 5c T = 2.5 s. We also used comsol in the case of thermal expansion of PDMS when subjected to the Joule heat generated by the liquid metal, and the results are shown in Figure S3 and the Supplementary Material Video S4. In addition to observing the evident thermal expansion of PDMS that causes the fracture of the liquid metal in Figure 5c, we have also ruled out two possibilities for the fracture of the liquid metal microheater: thermal expansion due to trapped air and electromigration. Firstly, we can exclude the reason for thermal expansion due to trapped air because a cavity is initially present in the microheater as shown in Figure 6b, and we do not observe an increase in this cavity during the microheater from the beginning of heating to fracture, which proves that the textured structure acts as a good exhaust. The reason for excluding electromigration is that the microheater shown in Figure 6b exhibits a noticeable necking region in its initial state. If the fracture of the microheater were caused by electromigration, it would occur at the necking region with the highest current density. However, as shown in Figure 6c, we did not observe such a fracture phenomenon in the necking region, and thus, we can exclude this possibility as well. Furthermore, we observed the texture substrate after the fracture of the microheater, as shown in Figure 6d. The textured structures on both sides of the channel have been damaged and fractured due to the high temperature of 300 °C, rendering them ineffective as exhaust channels. This also indicates that the PDMS with textured structures has reached its temperature limit. In conclusion, we believe that the main causes of fracture in the liquid metal microheater with textured structures are as follows: (1) PDMS undergoes significant thermal expansion during the heating process, making the original liquid metal in the channel unable to fill the entire channel at the high temperature of 300 °C and (2) when the heating temperature reaches around 300 °C, the textured structures on the microheater substrate film are severely damaged, unable to exhaust the air between the PDMS channel boundary after thermal expansion and the liquid metal inside the channel. The combination of these two reasons eventually leads to the fracture of the liquid metal microheater.

### 4.4. Stretching and Bending Test

After confirming the excellent heating performance of the microheater with a textured structure, we tested whether the microheater has a good flexibility. To ensure stable fixation on the testing platform, we fabricated a microheater with dimensions of approximately 6 cm × 3 cm (length × width) and an internal liquid metal heating wire length of approximately 70 cm.

**Figure 7:**
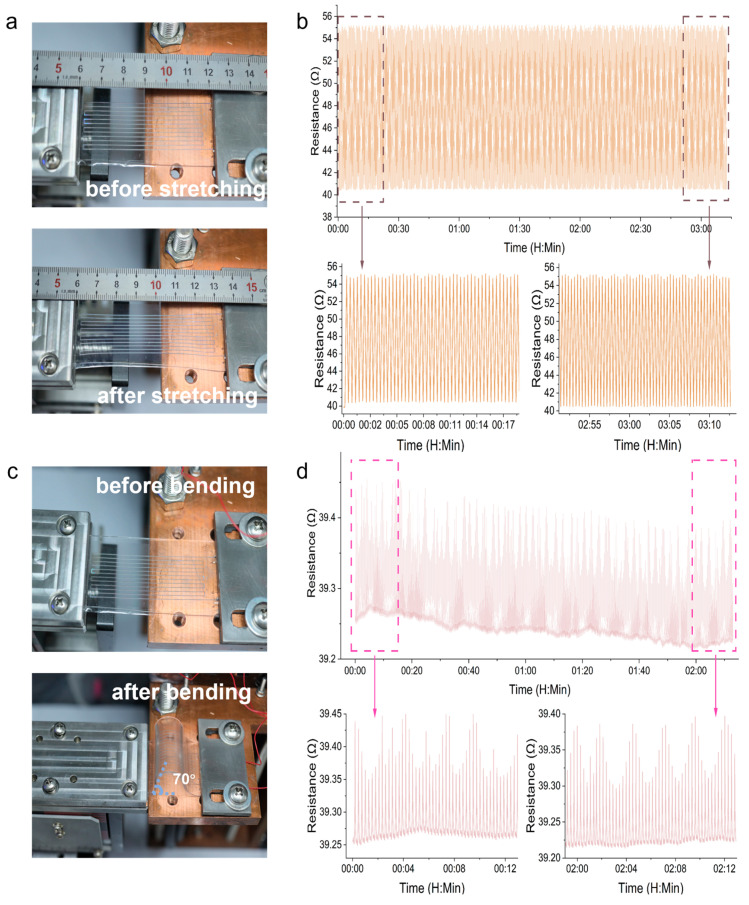
Mechanical flexibility testing of the microheater. (**a**) Microheater before and after stretching. (**b**) Resistance changes during 500 stretch cycles. (**c**) Microheater before and after bending. (**d**) Resistance changes during 500 bend cycles.

Firstly, we tested 500 tensile cycles on the microheater. Considering the stretching limit of PDMS itself, we set the stretching ratio to 20% (i.e., the displacement per single stretching cycle is 1.2 cm)—the physical appearance of the microheater before and after stretching is shown in Figure 7a. The variation of resistance over time during the stretching cycle is depicted in Figure 7b. From the graph, we can observe that the resistance changes steadily between 40 Ω and 55 Ω throughout the cycling process. This is because the liquid metal wire is elongated during stretching. According to Equation (2), the resistance of a conductor increases when its length is increased (R denotes the resistance, ρ represents the resistivity of the conductor, L denotes the length of the conductor, and A represents the cross-sectional area of the conductor). By comparing the resistance values of the first 50 cycles with those before the end 50 cycles, we can see the resistance values remain almost unchanged before and after stretching so that there is no fracture or leakage in the liquid metal wire during the stretching process.

(2) $$R=\rho \frac{L}{S}$$

Figure 7c shows the physical appearance of the microheater before and after bending. We nearly folded the microheater, resulting in a bending angle of approximately 70°. Figure 7d illustrates the variation of resistance over time during 500 cycles of bending. It can be observed that the resistance of the microheater exhibits periodic changes during the bending cycles, with a difference of approximately 0.07% between the beginning and the end of each cycle, which can be considered negligible.

Based on the aforementioned stretching and bending experiments, we can conclude that the liquid metal microheater exhibits excellent mechanical flexibility. It can be bent, stretched, and even folded without affecting its performance.

### 4.5. Application

**Figure 8:**
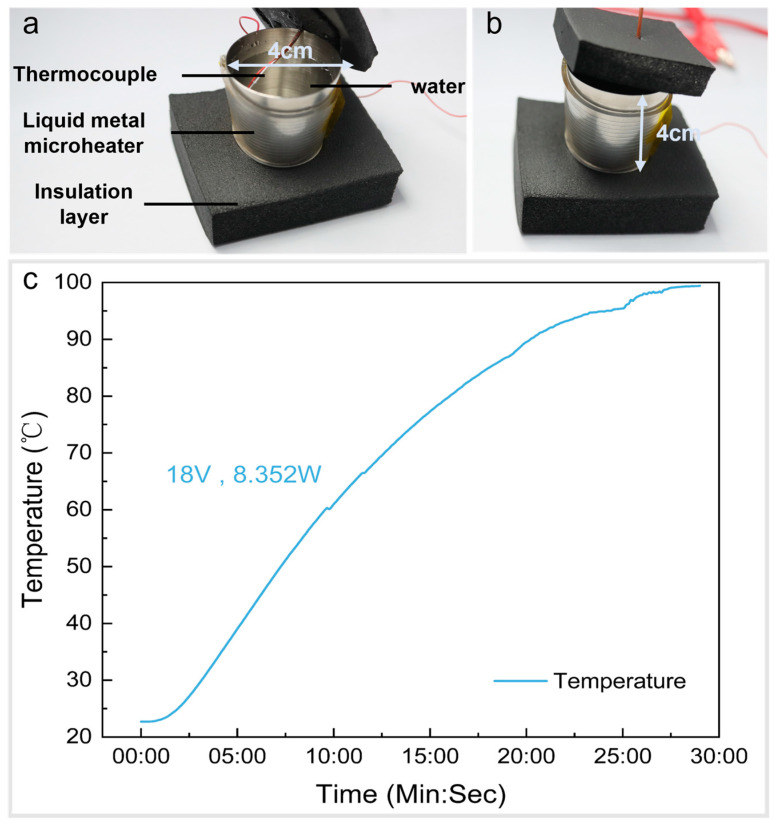
Schematic of water boiling device: (**a**) lift the upper insulation layer and (**b**) cover the upper insulation layer. (**c**) Temperature curve of water with time.

To demonstrate the good heating properties and mechanical flexibility of the microheater, the heater was attached to the side wall of a stainless steel cup to heat the water inside, as shown in Figure 8a,b. The cup is approximated as a cylindrical shape with a base diameter of 4 cm and a height of 4 cm. To prevent heat loss, insulation materials were added to the bottom and top of the cup, and a standard K-type thermocouple was probed into the cup from the top to measure the real-time temperature of the water. We applied a voltage of 18 V (I = 0.494 A, *p* = 8.892 W) to the microheater and heated about 50 mL of water in the cup from room temperature to boiling in less than 30 min, and the temperature curve of the water with time is shown in Figure 8c. Supplementary Material Video S5 shows what happens when the microheater boils water. According to Equation (3), the heating rate to boil water can be obtained as 2.9 °C/min (T is the temperature at which the water boils, T_0_ is the initial temperature of the water, and t is the heating time). From the formula for the amount of heat absorbed and the formula for the Joule heat, the formula for the thermal efficiency η of the microheater for heating water can be expressed as Equation (4), where c represents the specific heat capacity of water, m is the mass of water, and P is the heating power of the heater. The heating efficiency is calculated to be about 90%.

(3) $$K=\frac{T-T_{0}}{t}$$

(4) $$\eta =\frac{cm(T-T_{0})}{Pt}\times 100\%$$

These experimental results show that this flexible liquid metal microheater has great potential for use in portable or even wearable devices with heating needs.

## 5. Conclusions

In this study, a novel flexible liquid metal microheater with a textured structure is proposed. This microheater consists of a two-layer structure. The upper layer consists of a central liquid metal heating channel and parallel exhaust channels on both sides. The lower layer is a thin film with a textured structure. Through experiments on microheaters with and without the textured structure, it was found that the operating temperature of the liquid metal microheater could be increased substantially, and the maximum temperature difference between the two was greater than 200 °C. Furthermore, the microheater with the textured structure was compared to a microheater with parallel ventilation side channels made by Zhang et al. [30]. Although both used the principle of designing a venting structure to improve the performance of the microheater, we found from the experimental results that this microheater with a textured structure also had a significant increase in the maximum operating temperature of about 100 °C compared to their microheater. To explain the reasons for the performance improvement of the microheater and the eventual rupture of the liquid metal, a texture microheater with a length of only 6 mm was made for observation under a microscope. This allowed for the observation of the entire process of liquid metal fracture during the heating process. Based on the experiment, the following hypothesis was proposed: more densely distributed and numerous exhaust channels in the textured structure provided better ventilation performance, resulting in a higher maximum operating temperature. The final fracture of the liquid metal was primarily attributed to two reasons: (1) thermal expansion of PDMS and (2) the damage of the textured structure due to high temperature. The combination of these two factors ultimately led to the entrapment of a large amount of air inside the liquid metal channel, resulting in its fracture. This indicates to some extent that the current performance of liquid metal microheaters is mainly limited by the nature of the main material, i.e., PDMS itself. Several research studies have demonstrated that doping nanoparticles into PDMS can effectively improve its mechanical properties. For example, the doping of carbon nanotubes can effectively increase its elastic modulus [33], and the doping of metal nanoparticles can improve its Young’s modulus [34,35]. We believe that this kind of composite material based on PDMS is also very likely to be able to modulate the PDMS CTE, which will be the future research direction of liquid metal microheater based on microfluidic technology. Additionally, the tensile and bending characteristics of the microheater were tested. The experimental results demonstrated that the microheater exhibited good mechanical flexibility, and the liquid metal did not leak during repeated stretching and bending processes. Finally, the microheater was attached to the side wall of a cup for boiling water. It was found that the microheater could heat water from room temperature to boiling within 30 min. Based on the above, we believe that this liquid metal microheater, made with a textured structure, which exhibits an excellent mechanical flexibility and a significantly high operating temperature, has tremendous potential for applications in future portable and even wearable devices that require heating.

## Figures and Tables

**Table 1:** Comparison between six kinds of microheaters.

Width of Channels (µm)	Bottom Film Type	Highest Temperature (°C)	Corresponding Voltage (V)
200	Parallel straight texture	314.2	9.2
	Square texture	303.1	8.4
	No texture	115.5	4.4
400	Parallel straight texture	303.8	6.4
	Square texture	298.7	6.8
	No texture	72.1	2.4

## Data Availability

Data is contained within the article (and Supplementary Materials).

## Supplementary Materials

The following supporting information can be downloaded at: https://www.mdpi.com/article/10.3390/mi15010075/s1, Video S1: Two-dimensional simulation of temperature field variations in the microheater, Video S2: Infrared imaging of the chip during the heating process, Video S3: Liquid metal heating wire fracture process, Video S4: 3D simulation of PDMS thermal expansion, Video S5: Phenomena when water is heated to boiling using a liquid metal microheater; Figure S1: The x-section of microheaters on two textured substrates under the SEM: a. square texture. b. parallel straight texture; Figure S2: Real-time temperature and voltage profile of microheater; Figure S3: Three-dimensional electro-thermo-mechanical steady simulation results of microheaters. a. potential distribution on the microheater. b. temperature field distribution on the microheater. c. volume changes in microheaters due to thermal expansion of PDMS d. stress distribution on microheaters.
