# Supplementary material for: A Novel Flexible Liquid Metal Microheater with a Textured Structure
Source: Micromachines (Basel). 2023 Dec 29;15(1):75. doi: 10.3390/mi15010075 (PMC10818653; doi:10.3390/mi15010075)
Supplement: Supplementary file 1 [file micromachines-15-00075-s001.zip › Supplementary Information.pdf]

A novel flexible liquid metal microheater with a textured structure

Yuqing Li [1,2], Huimin Zhang [1,3], Zi Ye [1], Mingyang Liu [4], Wei Liu [4], Zhenming Li [4] and Lin Gui [1,2,*]

1 Key Laboratory of Cryogenic Science and Technology, Technical Institute of Physics and Chemistry, Chinese Academy of Sciences, 29 Zhongguancun East Road, Haidian District, Beijing 100190, China

2 School of Future Technology, University of Chinese Academy of Sciences, Beijing 100039, China

3 School of Engineering Science, University of Chinese Academy of Sciences, Beijing 100039, China

4 Energy Storage and Novel Technology of Electrical Engineering Department, China Electric Power Research Institute, Beijing 100192, China

* Correspondence: lingui@mail.ipc.ac.cn; Tel.: +86-010-82543483

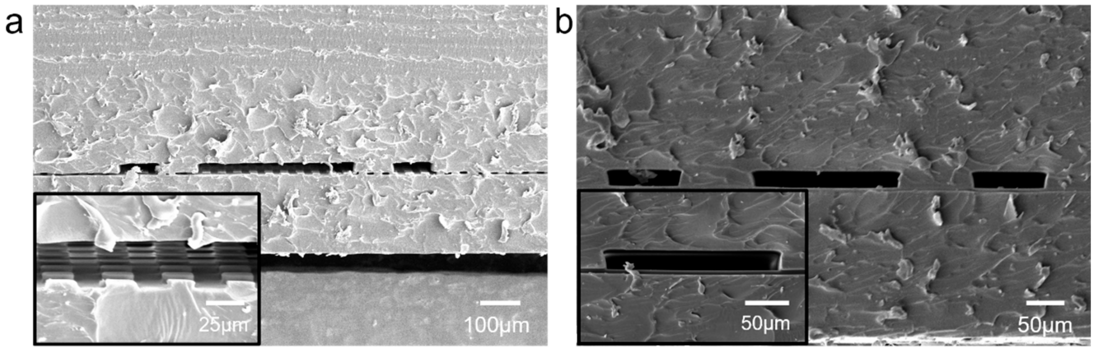

Figure S1. The x-section of microheaters on two textured substrates under the SEM: **a.** square texture. **b.** parallel straight texture.

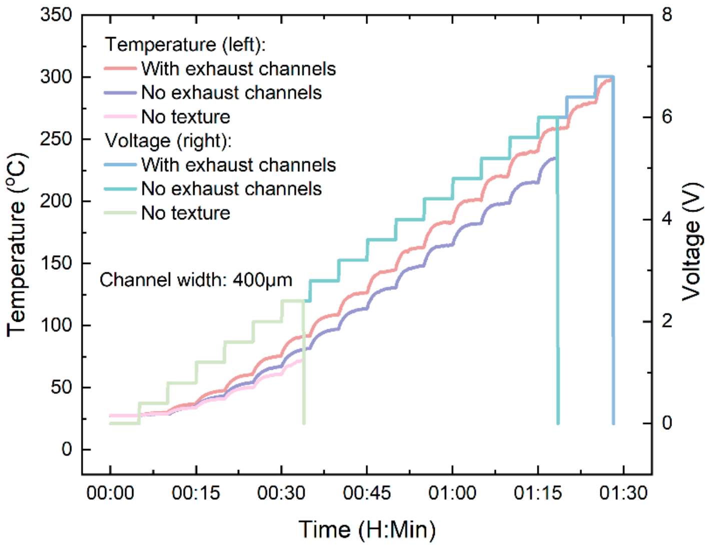

Figure S2. Real-time temperature and voltage profile of microheater.

Figure S2 shows the voltage and temperature versus time curves for the novel liquid metal microheater with and without side exhaust channel and a conventional microheater without a texture structure (channel width: 400 μm). From the figure, we can see that the performance of the microheater can be improved with or without the side exhaust channels, but the presence of the exhaust channel makes the performance improvement better.

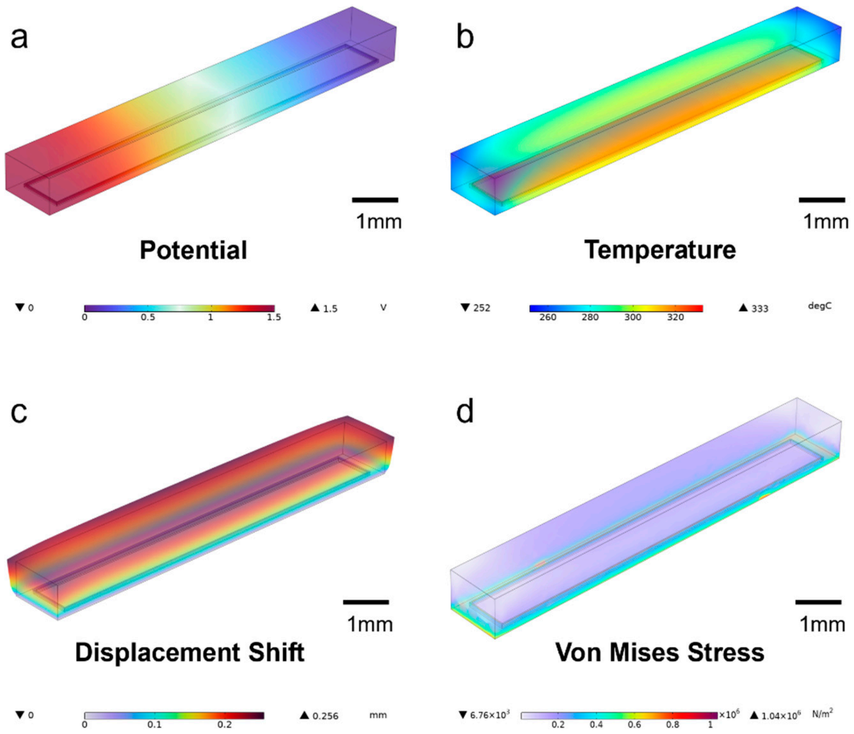

Figure S3. Three-dimensional electro-thermo-mechanical steady simulation results of microheaters. **a.** potential distribution on the microheater. **b.** temperature field distribution on the microheater. **c.** volume changes in microheaters due to thermal expansion of PDMS **d.** stress distribution on microheaters

Figure S3 illustrates the results of an electro-thermo-mechanical steady-state simulation of the heating process of a section of PDMS filled with liquid metal. Figures S3 a-d show the potential distribution, temperature distribution, PDMS volume change, and stress distribution on the PDMS surface after the system reaches the steady state, respectively. From the simulation results, it can be seen that the PDMS shows an obvious thermal expansion phenomenon when it reaches a high temperature of nearly 300 ℃, and the stress at the interface location between the PDMS and the liquid metal is large, which is very easy to cause damage to the chip.
